# Pharmacognostic characterization, wound healing and toxicity assessment of the stem bark of *Xylia evansii* Hutch (Leguminosae)

**DOI:** 10.1016/j.heliyon.2023.e21692

**Published:** 2023-10-26

**Authors:** Lord Gyimah, Evelyn Asante-Kwatia, Silas Adjei, Frederick Akuffo Owusu, Fanny Darko, Ernest Tabiri, Abraham Yeboah Mensah

**Affiliations:** a Department of Pharmacognosy, Faculty of Pharmacy and Pharmaceutical Sciences, College of Health Sciences, Kwame Nkrumah University of Science and Technology, Kumasi, Ghana; b Department of Herbal Medicine, Faculty of Pharmacy and Pharmaceutical Sciences, College of Health Sciences, Kwame Nkrumah University of Science and Technology, Kumasi, Ghana; c Department of Pharmaceutics, Faculty of Pharmacy and Pharmaceutical Sciences, College of Health Sciences, Kwame Nkrumah University of Science and Technology, Kumasi, Ghana

**Keywords:** *Xylia evansii*, Wound, Safety, Histopathology, Microscopy

## Abstract

*Xylia evansii* is widely used in traditional medicine to stop bleeding gums and treat wounds. This study was undertaken to assess the wound healing activity and toxicity profile of the stem bark methanol extract of *X. evansii* (XES). Wound healing activity was determined by the dermal excision model in rats. The free radical scavenging capacity, antioxidant activity, total phenolic and flavonoid contents were evaluated by the 1, 1-diphenyl-2-picrylhydrazyl (DPPH) free radical scavenging, total antioxidant capacity (TAC), aluminum chloride colorimetric and Folin Ciocalteu methods respectively. Acute and sub-acute oral toxicity assessment was performed following the Organization for Economic Co-operation and Development guidelines. Signiﬁcant (*p < 0.05*) dose-dependent wound healing effect, similar to that of 1 % silver sulphadiazine was elicit by the 10, 15 and 20 %^w^/_w_ XES ointments. The highest effect was demonstrated by XES 20 %^w^/_w_ which resulted in 98.3 % wound surface closure by day 9 of treatment (*p < 0.0001*). The total phenolic and ﬂavonoid contents were determined to be 381.2 ± 12.57 mg/g gallic acid equivalent (GAE) and 460 ± 29.07 mg/g quercetin equivalent respectively. XES exhibited remarkable free radical scavenging effect (IC_50_ = 68.13 ± 1.87 μg/mL) and had a total antioxidant capacity of 279.2 ± 32.08 mg/g GAE. The LD_50_ of XES was estimated to be > 5000 mg/kg. In sub-acute toxicity, 28 days treatment with XES (250, 500, 1000 mg/kg body weight) did not result in any significant (*p > 0.05*) change in the body weight or weight of the heart, lung, spleen, liver and kidneys. The haematological and biochemical profiles of XES-treated rats were not significantly (*p > 0.05*) affected after 4-weeks treatment with XES, except for platelet count which increased significantly (*p < 0.0001*) in a non-dose-dependent manner. Histopathological examination did not reveal any toxic effect to liver cells, however at 1000 mg/kg XES, slight abnormalities were identified in the glomeruli. Microscopy of the powdered stem bark displayed calcium oxalate crystals, pitted vessels and lignified fibres. Tannins, flavonoids, coumarins, saponins, triterpenes and alkaloids were identified in the bark. This is the first report on the wound healing potential and safety profile of *X. evansii*, giving scientific credence to its use in traditional medicine.

## 1 Introduction

Medicinal plants have been used for centuries to provide curative effects to several diseases. Herbal Medicine remains the most accessed therapeutic approach to a number of ailments especially in poor regions of the world [1]. In rural communities of African countries like Ghana, the use of medicinal plants in the management of common ailments is wide-spread due to their efficacy, availability and affordability [2,3]. Wound care is one of the most common conditions for which people resort to plants for healing [4]. In many African countries, inaccessibility to health centers and cost of medical care, are barriers to seeking early medical intervention for treatment of both acute and chronic wounds [5]. Indigenous herbs with wound healing effect are usually the main recourse for treatment of wounds in remote areas. Several medicinal plants and plant products have demonstrated the ability to instantly stop bleeding and promote healing of open wounds at a remarkably faster rate than their conventional counterparts [[6], [7], [8]]. In spite of the current technological advances in the field of orthodox medicine, investigations on alternative wound-healing agents especially from plant sources have occupied researchers from diverse parts of the world [9]. This is because, the diversity of plant secondary metabolites allows for their synergistic action in stimulating rapid wound healing through anti-inflammatory, antioxidant, antimicrobial, and other regenerative properties [[10], [11], [12], [13]].

The Ghanian flora provides a ready source of therapeutics for the local population. Previous ethnobotanical studies in Ghana, provides an extensive list of medicinal plants used by the local folks for healing both acute and chronic wounds [5,6,8]. *Xylia evansii* (family Leguminosae), is a plant known for its haemostatic and wound healing effect in Ghana. The plant grows in evergreen or semi-deciduous forests, along small watercourses and on hillsides with deep soils. It has a spreading crown and grows up to about 28–35 m tall [14]. The twigs of *X. evansii* are chewed to stop bleeding gums and heal mouth ulcers. The stem bark decoction is also used as cholagogue, hepatoprotective, wound healing and a general tonic [15]. Its fruits and seeds are a delicacy in some parts of Africa [14]. Plants of the genus *Xylia* are reported to be astringent and rich in tannins. In previous studies *Xylia* species were reported to possess antimicrobial [16], antioxidant [17], memory recovering, anticholinesterase [18], anti-inflammatory and analgesic activities [19]. Phenolic compounds including myricitrin and methyl gallate were identified in *Xylia xylocarpa* [17]. There is currently no scientific report on biological activities of the *X. evansii* despite its medicinal benefits in traditional medicine.

In a continuing effort to identify Ghanaian plants with substantial prospects for development of local wound healing agents [20,21], this study focused on evaluating the wound healing property of the stem bark of *X. evansii*. Since wound healing requires a delicate balance between oxidative stress and antioxidants [22], this study also evaluated antioxidant activity, total phenolic and flavonoid contents of the stem bark extract of *X. evansii*. To ascertain the safety level of the plant, acute and sub-acute oral toxicity studies were also conducted in animal models. Finally, some pharmacognostic and physicochemical parameters of the plant were established to aid in its identification and quality control [23]. This is the first report on scientific investigation of wound healing activity, toxicological assessment and pharmacognostic characteristics of *X. evansii*. Addition of such substantial value to locally used herbs makes them suitable candidates for future drug development.

## 2 Material and methods

### 2.1 Plant collection

*X.evansii* stem bark was collected from Kwahu-Asakraka in the Eastern Region of Ghana in November 2021. The authenticity of the plant materials was confirmed by Mr. Clifford Asare, Herbal Medicine Department, KNUST, Kumasi. A voucher specimen with number KNUST/HMI/2021/SB004 was kept at the Faculty of Pharmacy and Pharmaceutical Sciences (FPPS), KNUST herbarium.

### 2.2 Processing and preparation of plant extracts

The freshly harvested stem bark (1 kg) was garbled to remove all foreign materials and dried under a shade for two weeks. The dried sample was crushed into coarse powder with a mechanical grinder and kept in plastic bags at room temperature.

For biological activity screening, 400 g of the powdered sample was cold macerated with 1000 mL of methanol for 3 days. The extract obtained was reduced to a smaller volume by evaporating on a rotary evaporator at 55 °C under reduced pressure. The dried extract was weighed and its percentage yield determined as 5.19 %^w^/_w_. The extract labelled as ‘XES’ was kept in an air-tight glass container and stored in the fridge until required for use.

### 2.3 Phytochemical screening

Preliminary phytochemical screening was carried out on the powdered stem bark according to previously established methods [24].

### 2.4 Wound healing activity of XES

#### 2.4.1 Experimental animals

The Guidelines for the Care and Use of Laboratory Animals (Directive 2010/63/EU; Animal Care and Use Committee, 1998) were followed in all experiments involving animals. The Department of Pharmacology, Faculty of Pharmacy and Pharmaceutical Sciences, KNUST Ethics Committee approved all protocols used (FPPS-AEC/CA017/19). Male and female albino rats (14–20 g) were purchased from the Noguchi Memorial Institute for Medical Research (NMIMR), University of Ghana, Accra, Ghana. All animals were kept in steel cages (30 × 54 × 35 cm^3^) at the vivarium of the Pharmacology Department, KNUST. Ambient conditions i.e., temperature 25 ± 1 °C, relative humidity 60–70 %, under a 12 h dark-light cycle was maintained in the vivarium. The animals were allowed access to commercial rat pellets (GAFCO GH) and water *ad libitum*. Animals were allowed to familiarize with the laboratory environment for 7 days prior to the study.

#### 2.4.2 Acute dermal toxicity

The Guideline No. 402 of The Organization for Economic Co-operation and Development (OECD) (OECD, 2017) was employed to determine the acute dermal toxicity of the extract ointment. This was to ensure that the highest dose of extract ointment to be used for the wound healing assay would not be hazardous or detrimental to the animals following short-term exposure by the dermal route. Accordingly, an initial dose of 20 %^w^/_w_ of extract ointment was applied to the clean-shaven dorsal region of 4 female rats and observed for 24 h. As no signs of toxicity was observed following this application, 20 %^w^/_w_ extract ointment was considered to be safe for the wound healing study [25,26].

#### 2.4.3 Cream preparation

Three hundred grams (300 g) of emulsifying ointment was prepared according to a standard formula from the British Pharmacopoeia as shown on Table 1 [27]. Subsequently, three different concentrations i.e., 10 %^w^/_w_, 15 %^w^/_w_ and 20 %^w^/_w_ of XES cream, was prepared from the emulsifying ointment (Table 2). Four weeks following the formulation of the XES creams, the texture, odour and colour were closely monitored for any unexpected physical changes like separation, color or texture change.

**Table 1:** Formula for preparing emulsifying ointment.

Ingredients	Master Formula/g	Scale Formula/g (x 0.3; g)
Emulsifying Wax	300	90
White Soft Paraffin	500	150
Liquid paraffin	200	60

**Table 2:** Formula for aqueous cream preparation.

Ingredients	Master Formula	Scaled Quantities/g (x 0.0525)		
		Cream Concentration		
		10 %	15 %	20 %
**Emulsifying Ointment**	300g	15.75	15.75	15.75
**Chlorocresol**	10 ml	0.525	0.525	0.525
**Purified Water to**	1000 ml	52.5	52.5	52.5
**Extract**	105g	5.25	7.875	10.5

Amount of cream extract to prepare = 52.5g

#### 2.4.4 Animal grouping

For the excision wound experiment, 25 male albino rats were grouped into five (5 mice/group) as follows: the first group was treated with the cream base i.e., emulsifying ointment (negative control); the second group received topical application of 1 % silver sulphadiazine cream (positive control); groups 3, 4 and 5 were treated with 10 %^w^/_w_, 15 %^w^/_w_ and 20 %^w^/_w_ concentrations of the XES cream.

#### 2.4.5 Dermal excision wound model

Pentobarbitone 40 mg/kg body weight (bw) was used to anesthetize the rats by subcutaneous injection. A circular diameter of 50 mm was marked on the clean-shaven dorsal region of rats. The area was cleaned with 70 % ethanol before the excision wounds were made. The wounds were created by removing approximately 25–35 mm^2^ full thickness of the marked area on the anterior-dorsal region of each rat using toothed forceps, surgical blades and pointed scissors. The wounds were dressed with normal saline and the corresponding formulations were applied to the wound surfaces every 24 h for 21 days, beginning from the day of excision [7].

#### 2.4.6 Wound healing evaluation parameters

The wound surface diameter was measured with digital calipers every other day beginning from the day of wound excision till the 21st day of treatment. The wound surface area was then determined in mm^2^ as follows:

$wound\;surface\;area\;\left(WSA\right)=4\pi r^{2}$; where ***r*** is the radius of the wound**.**

The % wound surface closure was then determined by the formula:

$$\%\;wound\;surface\;closure=\frac{WSA\;\left(day\;1\right)-\;WSA\;\left[day\;n\right]}{WSA\;\left(day\;1\right)}\;x\;100$$

Where ‘*n’* represents other days of treatment.

### 2.5 Antioxidant activity of XES

#### 2.5.1 2, 2-diphenyl-1-picrylhydrazyl (DPPH) free radical scavenging assay

The radical scavenging ability of XES extract (31.25–500 μg/mL) was determined following a previous method [28]. Gallic acid (6.25–100 μg/mL) served as the reference substance and MeOH was used as the negative control. The experiments were performed three times and the mean of the three values determined. The percentage radical scavenging activity was determined by the formula:

$$\%\;DPPH\;scavenging\;activity=\frac{Abs\;control-\;Abs\;sample}{Abs\;control}\;x\;100$$

Where ‘Abs sample’ and ‘Abs control’ are absorbance of sample and control respectively. A graph of log concentration against percentage scavenging activity was plotted to generate an IC_50_ for the extract and gallic acid.

#### 2.5.2 Total antioxidant capacity (TAC)

The total antioxidant capacity was determined by a previously established method using gallic acid as the reference substance [29]. A standard line graph of concentration of gallic acid (6.25–100 μg/mL) against their respective absorbances was developed and the TAC of the extract was extrapolated from the line equation [y = 0.007411x +0.1280 R^2^ = 0.961]. TAC was given as gallic acid equivalent (GAE) in mg/g of dried extract [29].

#### 2.5.3 Total phenolic content (TPC)

The total phenolic content (TPC) of XES extract was determined by the Folin-Ciocalteu method [30] using gallic acid (6.25–100 μg/mL) as the standard. The TPC of the extract was extrapolated from the equation of the line, [y = 0.001017x + 0.05367, R^2^ = 0.9400] obtained from a standard line graph of gallic acid. TPC was given as gallic acid equivalent (GAE) in mg/g of dried extract.

#### 2.5.4 Total flavonoid content (TFC)

The total flavonoids in the crude extract was determined by the aluminum chloride colorimetry method, using quercetin as the reference substance [31]. A standard line graph of quercetin was developed and the TFC of the extract was extrapolated from the line equation [y = 0.007217x + 0.03460 R^2^ = 0.9954]. TFC was expressed as quercetin equivalent (QCE) in mg/g of dried extract.

### 2.6 Toxicity assessment of XES

#### 2.6.1 Acute oral toxicity

The acute oral toxicity of XES extract was assessed following the Limit dose test procedure as given by the Guideline 420 of the Organization of Economic Cooperation and Development (OECD) [32]. Albino rats of both sexes were randomly selected and put into two groups of five animals each. The animals were fasted overnight prior to the experiment. XES was solubilized in distilled water and a limit dose of 5000 mg/kg body weight (bw) was orally administered to animals once by gavage. The control group received 2 mL/kg of distilled water. The animals were then observed for mortality, general behaviour and any signs of toxicity (including diarrhoea, change in skin colour, change in fur, lacrimation, sedation, nostril discharge, salivation, ataxia, asphyxia, unusual locomotion, paralysis, lethargy, tremor or convulsion) continuously for an hour, then subsequently every 30 min for 4 h, then after 24 h and daily for 7 days, then on the 14th day. If three or more rats survived, the LD_50_ was stated to be greater than 5000 mg/kg body weight [33].

#### 2.6.2 Sub-acute oral toxicity

The sub-acute oral toxicity profile of XES extract was investigated following Guideline 407 of the Organization of Economic Cooperation and Development (OECD) [34,35]. The doses used were selected based on the LD_50_ and further calculations following the OECD guidelines. Twenty albino rats of both sexes were randomly selected and grouped into four (1–4) with 5 rats in each group as follows; Groups 1, 2 and 3 respectively received daily doses of XES at 250, 500, 1000 mg/kg body weight orally (*p.o.*) for 4 weeks and group 4 (negative control) received distilled water daily. Behavioural changes and other toxic signs as described in the previous section were monitored throughout the 28-day treatment period. The body weight of the animals was taken prior to study and subsequently, every week throughout the experiment and on the 28th day of study. After the 28-day period of treatment and observation, all animals were fasted overnight, anesthetized using halothane and euthanized on the 29th day by cervical dislocation. Heparinized and non-heparinized blood samples were collected in pairs for haematological and serum biochemical analysis.

##### 2.6.2.1 Hematological and biochemical analysis

An automated analyzer (BC-3000 Plus Auto Hematology Analyzer, Shenzhen Mindray Bio-Medical Electronics Co. Ltd, China) and a clinical biochemistry analyzer (Flexor Junior®, Vital Scientific, AC Dieren, Netherlands) were employed for the hematological and serum biochemical analysis respectively. Haematological parameters evaluated included: mean corpuscular volume (MCV), platelet count (PLT), platelet distribution width (PDW), platelet large cell ratio (P-LCR), red blood cell (RBC), haemoglobin (Hgb), white blood cell (WBC), procalcitonin (PCT), mean platelet volume (MPV), red blood cell distribution width (RDW_CV & RDW_SD), mean corpuscular haemoglobin (MCH), mean corpuscular haemoglobin concentration (MCHC) and hematocrit (HCT). Biochemical parameters analyzed included alanine aminotransferase (ALT), aspartate aminotransferase (AST), albumin (ALB), blood urea nitrogen (BUN), total protein (TP) and creatinine (CR) [36].

##### 2.6.2.2 Macroscopic inspection of internal organs and determination of organ weight

On the 29th day, internal organs including the liver, kidney, heart, spleen and lungs were removed, washed with 0.9 % NaCl and examined physically for any abnormality in gross morphology. The organs were then weighed using an electronic balance. The relative organ weights (ROW) were determined following a method described by Sellers et al. [37], as follows:

$$ROW=\frac{absolute\;organ\;weight\;\left(g\right)}{final\;body\;weight\;\left(g\right)}\;x\;100$$

##### 2.6.2.3 Histopathology study of the liver and kidney

The livers and kidneys of rats from all treatment groups and the negative control group, were preserved in 10 % buffered formalin and used for the histopathological analysis. Tissue sections were taken, dehydrated in ethanol and embedded in paraffin wax. Slides were prepared and stained with haematoxylin for the histopathological study [38]. Specimen were observed with the aid of an Olympus microscope (BX-51) and photographed by Infinity 4 USB Scientific Camera (Lumenera Corporation, Otawa, Canada).

### 2.7 Data analysis

All data are presented as the mean ± standard error of the mean (SEM). For the wound healing activity screening, comparisons were made between the negative control and treatment groups as well as the positive control group using the two-way analysis of variance (ANOVA) followed by Dunnet's *post hoc* test for multiple comparisons. Analysis of haematological and biochemical variations in sub-acute toxicity assay, were made between the negative control and the treatment groups by the one-way ANOVA followed by Dunnet's *post hoc* test. Analysis of variations in the body weight of rats in sub-acute toxicity assay was done by one-way ANOVA followed by Tukey's multiple comparison test. Results were considered statistically significant at *p* < 0.05. GraphPad Prism 6 for Windows (GraphPad Software, Inc.) was used for all analysis.

### 2.8 Pharmacognostic studies

#### 2.8.1 Organoleptic and macroscopic evaluation

The fresh stem bark of *X. evansii* was observed for organoleptic properties, bark surface features, fracture and curvature types (Evans, 2009).

#### 2.8.2 Micromorphological studies

The dried powdered stem bark was observed under a light microscope [DM-700 fitted with a camera, Leica ICC50 HD, Jos Hansen & Soehne Gmbh, Germany] for the presence of cell inclusions such as stones cells, calcium oxalate crystals, starches and other plant cell types (Evans, 2009). Photomicrographs were taken at × 10 and × 40 magnifications.

#### 2.8.3 Elemental content analysis

The presence and quantities of some essential minerals and metals in the stem bark of *X. evansii* was assessed by the Energy Dispersive X-ray Fluorescence [39].

#### 2.8.4 Physicochemical analysis

Physiochemical parameters such as solvent extractives, mineral content, total ash, water-insoluble ash and acid-insoluble ash were determined following standard methods [40].

#### 2.8.5 Infrared (IR) Fingerprinting

The IR spectra of the methanol extract was obtained from a PerkinElmer (model 1600) Fourier Transform-IR spectrophotometer.

## 3 Results

### 3.1 Preliminary phytochemical screening

The various classes of phytochemicals detected in the stem bark of *X. evansii* are presented on Table 3.

**Table 3:** Preliminary phytochemical screening of *X. evansii* stem bark.

Constituent	Test	Stem bark
Tannins	Ferric chloride test	+
Flavonoids	Alkaline reagent test	+
Alkaloids	Dragendorff's test	+
Coumarins	Fluorescence test	+
Reducing sugar	Fehling's test	+
Saponins	Frothing test	+
Triterpenoids	Salkowski's test	+
Phytosterols	Liebermann Buchard's test	–

(+) detected, (˗), not detected.

### 3.2 Wound healing activity

The odour, colour and texture of all formulated extract creams were not altered throughout the 4-week period of observation and usage. The pH remained fairly constant (7.2 ± 0.13) over the period. In the acute dermal toxicity assay, no sign of dermal toxicity (i.e., inflammation, irritation, or redness) was observed after 24 h of application of 20 %^w^/_w_ XES cream.

**Fig. 1:**
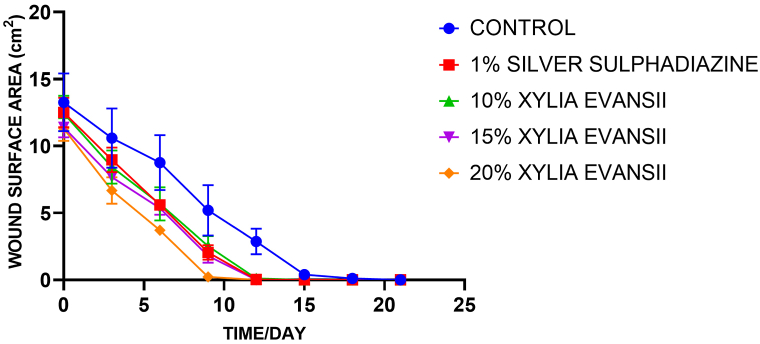

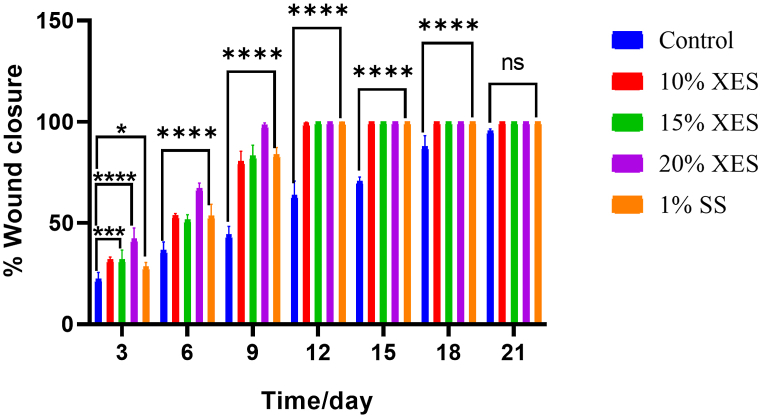
A: Time course curve of the wound surface area of *X. evansii* stem bark extract, standard and vehicle treated wounds over 21-day period of treatment [XES- *X. evansii* stem bark cream, SS- silver sulphadiazine].B: Effects of *X. evansii* stem bark extract on the percentage of wound closure area of excisional wound model. Each value is expressed as mean ± SEM (n = 5); SS- silver sulphadiazine; XES- *X. evansii* stem bark cream. Statistical analysis of % wound closure using two-way ANOVA followed by Dunnett's multiple comparison test; values were significantly different from negative control at ****p < 0.0001 (a), ***p < 0.001 (b), *p < 0.05 (c) and *p > 0.05* not significant (*ns)*.

Excisional wounds were created and treated daily with *X. evansii* extract cream and silver sulphadiazine cream (positive control) for 21 days. Fig. 1 displays a time course curve, showing the progression of wound healing, measured as the wound surface area in treated and untreated groups for 21 days. Table 4 and Fig. 1, present the mean percentage wound surface closures calculated for each group on the various days of treatment. A dose and time-dependent reduction in wound size was observed for all treatment groups. A significantly (*p < 0.0001*) rapid wound surface contraction was observed during the first nine days of treatment with XES (10, 15, 20 %^w^/_w_) and 1 % SS compared to the negative control group. A sharp decline in wound surface area was recorded between days 3 and 9. For all treated groups, ≥50 % wound closure was attained by day 6 which was significantly different (*p < 0.0001*) from the negative control group. XES 15 % and 20 % creams exhibited 100 % wound surface closure by the 12th day which was statistically significant (*p < 0.0001*) from the percentage wound surface closures recorded for the other treated groups. By day 15 of wound treatment, all extract- and 1 % silver sulphadiazine-treated groups had attained 100 % wound surface closure (*p < 0.0001*) while the rats treated with the ointment base (i.e., negative control group) had attained ∼ 70 % wound-surface closure. XES 15 % and XES 20 % exhibited a slightly faster rate of wound healing than 1 % silver sulphadiazine. Throughout the days of wound treatment, the rate of wound healing in the negative control group was slower than that of the XES and 1%SS-treated groups (Table 4).

**Table 4:** Percentage wound surface closure.

**Treatment** **Group**	**Percentage (%) wound closure**						
	Day 3	Day 6	Day 9	Day 12	Day 15	Day 18	Day 21
**10 % XES**	31.87 ± 1.24^b^	53.56 ± 1.01^a^	80.28 ± 5.14^a^	99.25 ± 0.46^a^	100^a^	100^a^	100^ns^
**15 % XES**	31.90 ± 4.71^b^	51.46 ± 2.61^a^	83.01 ± 5.35^a^	100^a^	100^a^	100^a^	100^ns^
**20 % XES**	41.97 ± 5.51^a^	67.00 ± 2.67^a^	98.26 ± 1.19^a^	100^a^	100^a^	100^a^	100^ns^
**1 % SS**	28.34 ± 2.1^c^	53.31 ± 5.92^a^	83.67 ± 3.51^a^	99.77 ± 0.1^a^	100^a^	100^a^	100^ns^
**Control**	22.17 ± 3.34	36.36 ± 4.24	44.03 ± 4.21	63.63 ± 7.06	70.53 ± 2.14	87.64 ± 5.46	95.44 ± 1.01

Each value is expressed as mean ± SEM (n = 5); SS- silver sulphadiazine; XES- *X. evansii* stem bark cream. Statistical analysis of % wound closure using two-way ANOVA followed by Dunnett's multiple comparison test; values were significantly different from negative control at ****p < 0.0001 (a), ***p < 0.001 (b), *p < 0.05 (c) and *p > 0.05* not significant (ns).

### 3.3 Antioxidant activity

The stem bark extract of *X. evansii* demonstrated remarkable concentration dependent DPPH free radical scavenging. The extract was also found to contain high phenolic and flavonoid content and exhibited remarkable total antioxidant capacity. The IC_50_ values for DPPH scavenging effect, total antioxidant capacity, total phenolic and flavonoid content are presented on Table 5.

**Table 5:** Antioxidant activities of *X. evansii* and stem bark.

Extract	DPPH (IC_50_/*μ*g/mL)	TAC (mg/g GAE)	TPC (mg/g GAE)	TFC (mg/g QCE)
XES	68.13 ± 1.87	27.92 ± 1.081	38.12 ± 1.57	460 ± 29.07
Gallic acid	18.92 ± 0.43			

XES- *X. evansii* MeOH stem bark extract.

### 3.4 Toxicity assessment

#### 3.4.1 Acute oral toxicity

In acute oral toxicity studies, a single dose of 5000 mg/kg body weight (bw) of *X. evansii* stem bark extract did not result in any sign of toxicity, physiological or behavioral changes in both control and treated groups at 30 min, 4 h, 24 h, 48 h, 72 h, 7 days and 14 days of observation. All the animals remained active and healthy and showed no abnormal change in their pattern of feed and water intake. No mortality was recorded within this period. The LD_50_ of *X. evansii* stem bark extract was determined to be above 5000 mg/kg body weight.

#### 3.4.2 Subacute oral toxicity study

In the sub-acute toxicity study, the rats were administered 250, 500 and 1000 mg/kg bw/day of XES for 28 days (four weeks). The control group received distilled water. Specific parameters including clinical signs of toxicity, physiological or behavioural changes, mortality, alteration in body and internal organ weights, haematological and biochemical indices were monitored. The liver and kidney were prioritized for histopathological assessment after the 28 days of treatment as these organs are known to be targets of several toxic chemical compounds due to the role they play in excretion and detoxification. Details of the results of subacute toxicity studies are given below.

##### 3.4.2.1 Clinical observation and mortality

No clinical signs and symptoms of toxic effect on behavioral or physiological patterns were recorded in XES treated rats. There was no change in the eyes, nose (nasal secretion), skin, fur, sleep pattern and salivation during the 28-day period of XES administration. Both treated and control groups appeared healthy at the end of the treatment period without any record of mortality. Food and water consumption as well as urinary and faecal excretion patterns of the treated rats were not significantly different from the negative control group.

##### 3.4.2.2 Weekly body weight

Weekly assessment of the body weights of both XES-treated and control groups showed a steady increase in body weight in all rats over the 4-week period of the experiment. This could be attributed to the normal growth pattern of the rats as well as the nutritive constituents of their feed. The increase in body weight for all groups was consistent weekly without any significant differences in percentage body weight gain between the treated and control groups. The weekly body weights as recorded on days 7, 14, 21 and 28 are presented on Table 6.

**Table 6:** The effect of *X. evansii* stem bark extract on weekly body weight of rats during 28-day treatment.

Groups	Dose	Body weights (grams)				
		1st day	7th day	14th day	21st day	28th day
1	Control	101.0 ± 3.033	102.2 ± 2.691	104.6 ± 2.561	107.2 ± 2.728	109.6 ± 3.043
2	250 mg/kg(bw)	108.4 ± 3.655	110.6 ± 3.487	113.6 ± 3.370	116.4 ± 3.356	119.0 ± 3.271
3	500 mg/kg(bw)	110.2 ± 3.426	111.6 ± 3.219	114.2 ± 3.338	118.4 ± 3.234	120.6 ± 3.219
4	1000 mg/kg(bw)	108.4 ± 1.778	110.4 ± 1.939	111.2 ± 1.772	115.6 ± 1.806	118.6 ± 1.833

Each value is expressed as mean ± SEM (n = 5). Statistical analysis of rat body weight using one-way ANOVA followed by Tukey's multiple comparison test; values were not significantly different from the negative control group (*p > 0.05*).

##### 3.4.2.3 Gross morphology of internal organs and relative organ weight

Macroscopic evaluation of the heart, lungs, spleen, kidneys and liver after 28 days of treatment with XES 250, 500 and 1000 mg/kg body weight, did not show any abnormality in organ structure, colour or presence of pathological lesions on organs when compared to the control groups. The relative organ weights of all treated and non-treated groups are presented on Table 7*.* There was no significant difference (*p > 0.05*) in the relative organ weight (organ-to-body weight ratio) of isolated organs between the XES-treated and the negative control group.

**Table 7:** Effect of *X. evansii stem bark extract* on relative organ weight of rats after 28-day treatment.

**Organs**	Relative Organ Weight (gram ± SD)			
	Control	250 mg/kg(bw)	500 mg/kg(bw)	1000 mg/kg(bw)
**Liver**	4.81 ± 0.47	4.43 ± 0.24	4.47 ± 0.19	5.15 ± 0.28
**Heart**	0.488 ± 0.028	0.480 ± 0.047	0.511 ± 0.021	0.503 ± 0.026
**Spleen**	0.570 ± 0.054	0.536 ± 0.021	0.615 ± 0.072	0.622 ± 0.024
**Lungs**	1.037 ± 0.090	1.364 ± 0.284	1.228 ± 0.143	1.361 ± 0.146
**Right kidney**	0.393 ± 0.021	0.401 ± 0.022	0.417 ± 0.017	0.412 ± 0.017
**Left kidney**	0.379 ± 0.018	0.389 ± 0.022	0.414 ± 0.017	0.399 ± 0.019

Each value is expressed as mean ± SEM (n = 5). Statistical analysis of relative organ weight using one-way ANOVA followed by Dunnett's post hoc test; values were not significantly different from the negative control group (*p > 0.05*).

##### 3.4.2.4 Haematological parameters

Table 8 shows the results of haematological assessment of rats after 4 weeks treatment with XES 250, 500 and 1000 mg/kg bw/day. From the results, XES at all doses did not cause any statistically significant (*p > 0.05*) alteration in all parameters analyzed compared to the negative control group except for platelets (PLT). A significant, but non-dose dependent increase in platelets (PLT) at 250 mg/kg bw (*p < 0.001*), 500 mg/kg bw (*p < 0.0001*) and 1000 mg/kg bw/day (*p < 0.01*) was recorded. This variation was however within the normal range of PLT for rats [33].

**Table 8:** Effect of *X. evansii* stem bark extract on haematological parameters of rats after 28-day treatment.

Parameters	Control	250 mg/kg(bw)	500 mg/kg(bw)	1000 mg/kg(bw)
WBC (x10^3^/μL)	13.28 ± 0.29	14.92 ± 2.27	12.89 ± 1.23	11.54 ± 0.77
RBC (x10^3^/μL)	5.76 ± 0.06	6.07 ± 0.31	5.83 ± 0.22	5.53 ± 0.49
HGB (x10^3^/μL)	13.70 ± 0.32	14.77 ± 0.01	14.70 ± 0.72	13.87 ± 0.82
HCT (%)	32.23 ± 0.49	35.23 ± 1.18	35.27 ± 1.24	32.37 ± 2.97
MCV (FI)	56.07 ± 1.47	58.23 ± 1.09	60.67 ± 0.30	58.63 ± 173
MCH (pg)	23.73 ± 0.81	24.43 ± 0.61	25.17 ± 0.54	24.53 ± 0.68
PLT (x10^9^/L)	470.0 ± 83.93	672.0 ± 37.24***	891.7 ± 228.9****	628.3 ± 121.2**
MCHC (g/dL)	42.33 ± 0.33	42.00 ± 0.00	41.67 ± 0.67	41.67 ± 0.88
RDW-CV (%)	19.07 ± 0.52	16.17 ± 0.78	16.23 ± 1.19	16.77 ± 0.45
RDW-SD	30.57 ± 087	30.30 ± 1.03	27.87 ± 3.62	31.13 ± 1.02
MPV (fl)	6.73 ± 0.23	6.67 ± 0.033	6.50 ± 0.15	6.73 ± 0.27
PDW (%)	8.23 ± 0.20	8.20 ± 0.00	7.98 ± 0.23	7.77 ± 0.30
PCT (ng/mL)	0.31 ± 0.05	0.44 ± 0.03	0.57 ± 0.13	0.41 ± 0.07
P-LCR (fl)	20.72 ± 2.73	14.26 ± 0.09	9.81 ± 1.31	14.36 ± 2.76

Each value is expressed as mean ± SEM (n = 3). Statistical analysis of haematological markers by the one-way ANOVA followed by Dunnett's post hoc test; values were significantly different from negative control group at (**p < 0.01) (***p < 0.001) and (****p < 0.0001). [**WBC**: white blood cells; **RBC**: red blood cells; **HGB**: hemoglobin; **HCT**: Hematocrit; **MCV**: Mean Corpuscular Volume; **MCH**: mean corpuscular hemoglobin; **PLT:** platelet, **MCHC:** mean corpuscular hemoglobin concentration**; RDW:** red cell distribution width; **MPV**: mean platelet volume; PDW: platelet distribution width; **PCT**: procalcitonin; **P-LCR:** platelet-large cell ratio].

##### 3.4.2.5 Biochemical analysis

The effect of *X. evansii* stem bark extract on some biochemical parameters after the 28-day treatment of rats are summarized on Table 9. From the results, plasma levels of enzymes for liver function (i.e., aspartate transaminase (AST) and alanine transaminase (ALT)) did not show any significant change for the groups treated with 250 and 500 mg/kg bw/day. Meanwhile, significant decrease in both AST (*p < 0.01)* and ALT (*p < 0.05)* were recorded in the groups treated with 1000 mg/kg bw/day of XES. No significant (*p > 0.05*) alteration was recorded in the levels of albumin and total protein. Parameters for kidney function, i.e., serum creatinine (CRT) and blood urea nitrogen (BUN) levels, did not show any statistically significant (*p > 0.05*) differences between the groups treated with 250, 500 and 1000 mg/kg bw/day of XES, compared to the control group (Table 9).

**Table 9:** Effects of *X. evansii* stem bark extract on biochemical parameters of rats after 28-day treatment.

Parameters	Control	250 mg/kg(bw)	500 mg/kg(bw)	1000 mg/kg(bw)
AST (μ/L)	102.8 ± 18.48	101.1 ± 12.56	74.60 ± 7.284	56.20 ± 11.87**
ALT (μ/L)	87.13 ± 10.41	85.40 ± 6.74	63.20 ± 12.53	47.83 ± 3.472*
TP (g/L)	74.50 ± 1.74	68.10 ± 1.08	79.60 ± 1.80	77.57 ± 1.63
ALB (g/L)	34.37 ± 6.24	31.60 ± 6.73	35.50 ± 1.88	45.47 ± 4.13
BUN (mmol/L)	3.13 ± 1.14	3.37 ± 1.59	4.80 ± 0.23	3.83 ± 0.12
CR (mmol/L)	69.33 ± 28.53	72.1 ± 8.532	68.8 ± 10.24	94.57 ± 7.337

Each value is expressed as mean ± SEM (n = 3). Statistical analysis of biochemical markers by the one-way ANOVA followed by Dunnett's post hoc test; values were significantly different from negative control group at (*p < 0.05), (**p < 0.01) [AST: aspartate aminotransferase; ALT: alanine transaminase; **TP**: total protein; **ALB**: albumin; **CR**: creatinine; **BUN:** blood urea nitrogen].

##### 3.4.2.6 Histopathological study

**Fig. 2:**
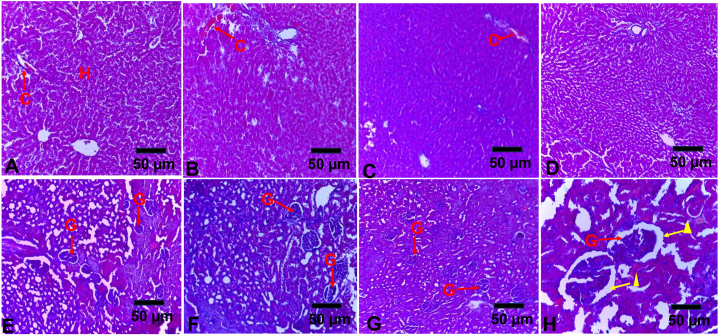
A–H: Photomicrographs of histopathological sections (x40 magniﬁcation) of the liver and kidney tissues stained with haematoxylin and eosin [Fig. 2A: liver of negative control group; Fig. 2B–D: liver of group treated with XES 1000, 500, 250 mg/kg respectively; Fig. 2E: kidney of negative control group, Fig. 2F–H: kidney of group treated with XES 1000, 500, 250 mg/kg respectively.

At the end of the 28-day treatment, the liver and kidneys were prioritized for histopathological assessment due to the role played by these organs in excretion and detoxification, which makes them a target of several toxic chemical compounds [34]. The histopathological examination of the rats’ livers showed normal hepatocellular architecture without any sign of abnormality between the control group and those treated with 250, 500 and 1000 mg/kg bw/day of XES. Hepatocytes radiating around the central vein were well preserved, devoid of inflammation, and there was no blood vessel congestion (Fig. 2; A-D).

From the photomicrographs of the kidney of the rats in the control and those treated with 250 and 500 mg/kg body weight/day of XES, the Bowman's capsules were preserved with no inflammatory cell infiltrations or blood vessel congestion. Podocytes and glomerular structures were well preserved (Fig. 2; E, F, G). However, a slight variation was observed in rats treated with XES 1000 mg/kg body weight/day where a slight expansion of the Bowmans space was seen (yellow triangle) (Fig. 2H).

### 3.5 Pharmacognostic studies

#### 3.5.1 Morphological description and microscopy of the stem bark powder of X. evansii

**Fig. 3:**
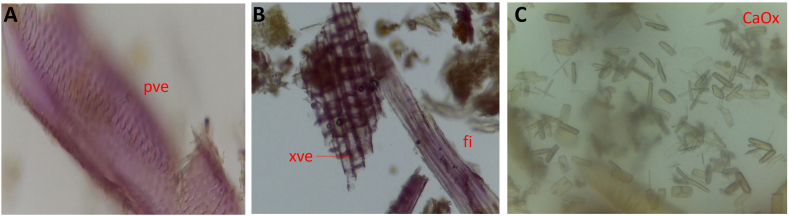
A–C: Microscopy of powdered stem bark of *X. evansii* displaying bundles of pitted xylem vessels, fibres, acicular and prismatic calcium oxalate crystals [**pve**-pitted xylem vessels; **xve**-xylem vessels; **fi**-fibres; **CaOx**-calcium oxalate crystal].

The fresh stem bark of *X. evansii* was greyish-brown on the outer bark with vertical striations and few mosses and the inner bark appeared light brown with streaks of dark brown coloration. The microscopy of the powdered stem bark showed the presence of pitted xylem vessels, acicular and prismatic calcium oxalate crystals and bundles of fibers (Fig. 3A–C).

#### 3.5.2 Physicochemical analysis

Physicochemical parameters evaluated for the stem bark of *X. evansii* are presented on Table 10.

**Table 10:** Physicochemical properties of *X. evansii* stem bark.

Parameter	Stem bark
Extractive values % (w/w)	
Water	19.27 ± 3.154****
Methanol	26.42 ± 3.106****
Ethyl acetate	10.34 ± 1.259****
Chloroform	10.01 ± 1.263****
Petroleum ether	8.56 ± 1.670****
Elemental content (mg/L)	
Iron (Fe)	18.03 ± 0.003
Lead (Pb)	0.019 ± 0.003
Zinc (Zn)	0.38 ± 0.003
Copper (Cu)	0.15 ± 0.002
Calcium (Ca)	1.29 ± 0.006
Potassium (K)	28.00 ± 2.45
Ash content (% w/w)	
Total ash	5.64 ± 1.032
Water soluble ash	5.21 ± 0.825
Acid - insoluble ash	0.60 ± 0.061

Values are presented as the mean ± standard deviation for three (3) replicates. Statistical analysis of % extractive values of different solvents using ordinary one-way ANOVA. Extractive values were significantly different from each other (****p < 0.0001).

#### 3.5.3 Infrared (IR) Fingerprinting

**Fig. 4:**
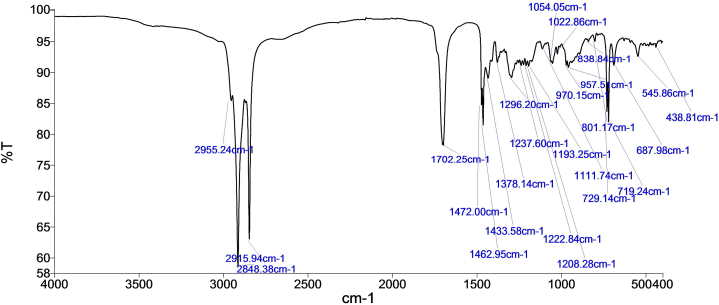
Infrared spectroscopy fingerprint of the MeOH extract of *X. evansii*.

A characteristic IR spectra of *X. evansii* stem bark (Fig. 4) was developed for the quality assessment of herbal preparations containing this plant sample. The IR spectra showed absorption bands mainly around 2800-2900-cm^−1^ (strong, sharp) for C–H stretching representing alkane or aldehyde groups and around 1700 cm^−1^ (sharp) for carbonyl group (C=O stretch).

## 4 Discussion

Studies regarding the biological activities and safety of medicinal plants are very important, as they are logical search strategies in the discovery of new drugs [33]. This study was undertaken to evaluate the wound healing property of the methanolic extract of the bark of *Xylia evansii* (XES), and assess its acute and sub-acute toxicity in rats. Further, some pharmacognostic and physicochemical parameters for identification and quality control purposes were established.

The excisional wound model was employed in the wound healing assay. This model is the most commonly used wound healing model, considered to resemble an acute clinical wound, which requires healing by secondary closure [41]. The method has been used in the assessment of the wound healing property of several plants [12,42,43]. It is the most predictive model for evaluating wound healing activity since it allows a realistic representation of the wound environment, enabling investigation of haemorrhage, inflammation, granulation tissue formation, re-epithelialization, angiogenesis and collagen biosynthesis [44]. In this method, the rate of wound healing can be easily determined by measuring the wound surface area over a specific time frame and compared against the original wound sizes [45].

From the results, topical application of the *X. evansii* extract ointment caused a remarkable increase in the rate of wound surface closure of open wounds compared to the negative control group. There was a noticeable positive response to treatment throughout the treatment period. By the 15th day of treatment, 100 % wound closure (*p < 0.0001*) was recorded for all rats treated with the extract ointments compared to 70.53 % for the group treated with the blank ointment. The overall results suggest that higher concentrations of XES cause significant increase in wound contraction within a short time. The methanol extract of *X. evansii* stem bark contained remarkable flavonoid (460 ± 29.07 mg/g QCE) and phenolic constituents (38.12 ± 1.57 mg/g GAE) and exhibited significant DPPH radical scavenging activity (IC_50_ = 68.13 ± 1.87). Previous studies have established a good correlation between antioxidant activity and wound healing [11,22]. This implies that the antioxidant activity of *X. evansii* stem bark may greatly contribute to its wound healing effect. Phytochemical constituents such as tannins, coumarins, triterpenoids and flavonoids, which were identified in *X. evansii*, are known to promote wound contraction and re-epithelialization [46], through their astringent [47], antioxidant [10], anti-inflammatory [12] and antimicrobial activities [44,48]. Plant constituents have been proven to promote contraction of membrane proteins, formation of new capillaries and fibroblasts and increase deposition of collagen at the wound sites [12,49].

In-spite of the widespread usage of plant medicines, investigations carried out on over 1.5 million plants, indicate that some medicinal plants contain toxic substances [50]. Prior to any drug discovery research, toxicity assessment is required to determine the preclinical doses for a plant's pharmacological validation and its safety [33]. Acute and subacute toxicity studies are conducted to provide information on the toxic potential of a medicinal plant based on short-term use [34]. In acute oral toxicity studies, the LD_50_ of *X. evansii* stem bark extract was determined to be more than 5000 mg/kg body weight, as rats did not show any sign of toxicity after treatment at this limit dose. Based on the Hodge and Sterner classification for toxicity [51], the stem bark of *X. evansii* could be classified as practically non-toxic.

Subacute toxicity studies enable the detection of toxic effects, which develop after administering repeated doses of a substance in the short term. It enables the detection of abnormal variations in body weight, haematological parameters and biochemical indices after a period of drug/extract administration [38]. From our results, all animals appeared well with no behavioral signs of systemic toxicity or death after the 28-day treatment period. The absence of significant reduction in the rats’ body weight indicates that the extract did not affect the appetite of the animals, or exert any depressive effect on cellular metabolism. The risk of damage to internal organs including the kidneys, liver, lungs, spleen and heart were assessed by physical examination as well as by the determination of the relative organ weights. Physical assessment of the heart, lungs, spleen, kidneys and liver showed no abnormality in organ structure, colour, or presence of pathological lesions on the major organs of rats treated with XES extracts compared to the negative control group.

The assessment of haematological parameters during toxicity studies is very critical, as alterations in haematological indices of rats have high predictive values for humans [38]. A rise or decline in a haematological variable could be a pointer to excessive or suppressed haematopoeisis, bone marrow function or immune function [52]. From the results, treatment with XES (250, 500,1000 mg/kg bw) did not result in any significant (*p > 0.05*) change in the rats’ haematological parameters apart from platelets which were markedly increased at 250 mg/kg bw (*p < 0.001*), 500 mg/kg bw (*p < 0.0001*) and 1000 mg/kg bw/day (*p < 0.01*) in a non-dose dependent manner. This irregular increase in platelet count may be as a result of circulating platelets, which were increased and mobilized to the site of injury due to inflammation or bleeding during blood collection [36,53]. On the hand, the extract may also have a stimulatory effect on thrombopoietin, causing an increased bone marrow production of platelets which could contribute to its use as a haemostatic in traditional medicine [54]. The fact that there was no significant (*p > 0.05*) alteration in parameters such as WBC, RBC, HGB, RDW, MCH and MCHC suggests that XES has no stimulatory or depressive effect on the haematopoietic or immune system. Moreover, the slight differences recorded were within physiologically acceptable limits [38]; probably representing peculiarities and individual variations in animals and not linked with a toxic effect of the extract.

The liver and kidneys play an essential role in the processes of excretion and detoxification. This makes these organs a target for several toxic chemical compounds [38]. There are frequent reports on the hepatoxic and nephrotoxic effects of traditional herbal medicines [52]. In biochemical analysis, parameters including serum alanine transaminase (ALT), aspartate transaminase (AST), total protein and albumin were assessed for liver function. Generally, an increase in plasma AST or ALT is an indicator of a type of cell damage to the hepatic, renal, cardiac or skeletal muscles, though a rise in ALT is more specific to hepatic damage. A decline in total protein and albumin is also indicative of diminished or impaired hepatocellular function [38,55]. From the results, it was observed that lower doses of XES (250–500 mg/kg bw/day) caused no alteration in the levels of AST and ALT compared to the negative control group. However, at 1000 mg/kg bw/day, a significant decrease in plasma levels of AST (*p < 0.01*) and ALT (*p < 0.05*) was recorded. Total protein and albumin levels were not significantly affected by all doses of XES extract. The observed decrease in AST and ALT together with the non-significant alteration in total protein and albumin levels suggest that the extract had no deleterious effect on hepatic cells. This was supported by the normal hepatocellular architecture after treatment as seen in histopathology.

High plasma creatinine and urea concentrations suggest a declined ability of the kidneys to filter these metabolic wastes from the blood [38]. In our study, blood urea nitrogen and creatinine levels were not significantly altered in all XES-treated groups compared to the control group. Meanwhile histopathological examination of the kidney showed a slight variation in kidney architecture of rats treated with XES 1000 mg/kg bw/day. This suggests a possible deleterious effect on glomeruli after prolonged use of the extract. However, taking into consideration the normal levels of biochemical markers of kidney function, further studies into the nephrotoxic effect of XES may be required to substantiate this observation. It must be taken into consideration, that the complexity of whole animal experiments, makes it difficult to account for the distinct contribution of individual immune responses of tissues and cells during in a biochemical or physiological reaction.

In the pharmacognostic study of *X. evansii,* lignified fibres, pitted xylem vessels and acicular/prismatic calcium oxalate crystals were identified in the microscopy of the powdered stem bark. From the extractive value determination, polar solvents such as methanol and water had higher extractive power and afforded higher yields than non-polar solvents. *X. evansii* bark had a total ash of about 5 %. This implies that the residual matter remaining after incineration of 2 g of dried powdered stem bark must be less than 10 %. The acid-insoluble ash which specifies possible adulteration with siliceous materials was less than 1 %^w^/_w_, meaning that the amount of inorganic matter naturally adhering to 2 g of powdered material should not exceed 0.02 g. Analysis of mineral content revealed appreciable amounts of essential minerals such as calcium, potassium, magnesium, phosphorus, zinc and iron which contribute to plant's role in the prevention and treatment of diseases [56,57]. The level of lead [Pb], which poses great threat to human health was below the lowest permissible limit i.e., 10 mg/kg, set by the Food and Agricultural Organization (FAO) [58]. Nonetheless, the geographical location, mineral composition of the soil, climatic conditions and human activities are factors that may influence the elemental content of any plant drug [58]. It is also worth noting that the health risk associated with the consumption of heavy metals is dependent on the average daily intake [57].

## 5 Conclusion

The study underscores the critical importance of bridging traditional wisdom with modern scientific exploration for the betterment of health and well-being, therefore, very important in its field. The study's comprehensive approach not only contributes to scientific knowledge but also has the potential to impact healthcare practices, traditional medicine validation, and ecological awareness. The results of this study have revealed that topical application of *X. evansii* stem bark extract to open wounds accelerates wound healing. Moreover, the plant is a potential source of natural antioxidants. Phytochemicals including tannins, saponins, flavonoids, coumarins, triterpenoids and alkaloids identified in the plant may contribute to its wound healing effects. Under the conditions tested, the LD_50_ of *X. evansii* stem bark extract is above 5000 mg/kg bw/day. The extract demonstrated no hepatotoxic or nephrotoxic potential at concentrations up to 500 mg/kg body weight/day. Inability to obtain a histopathological report on wound tissues of the various treated groups was a limitation of this study, as this could have given a better understanding on the influence of the extract on collagen synthesis/deposition and re-epithelization. Nevertheless, the significant reduction in treated wound sizes compared to the negative control group indicates an increased amount of extracellular matrix required for the repair of defective cells and re-epithelialization. Again, histopathological assessments of other internal organs i.e., heart, lungs and spleen are recommended in future toxicity studies. The results provide for the first time very relevant data on the wound healing potential and safety profile of *X. evansii* stem bark.

## Ethical considerations

All protocols regarding the use of experimental animals were approved by the Department of Pharmacology, Faculty of Pharmacy and Pharmaceutical Sciences, KNUST Ethics Committee approved all protocols used (FPPS-AEC/CA017/19).

## Funding statement

This work did not receive any funding from an external source.

## Data availability statement

The raw data/results from experiments used to arrive at the findings have been provided in this report. Other reports that were used to support this study are cited at relevant places within the text as references.

## Additional information

No additional information is available for this paper.

## Declaration of competing interest

The authors declare that they have no known competing financial interests or personal relationships that could have appeared to influence the work reported in this paper.
